# Neonatal Sacrococcygeal Teratoma: A Case Report

**DOI:** 10.31729/jnma.9090

**Published:** 2025-06-30

**Authors:** Riyaz Shrestha, Mohammad Adnan Adil, Swechha Gautam, Prajeet Ray, Rupesh Jung Raut, Dinuj Shrestha, Prayash Chand, Prakash Bista

**Affiliations:** 1 Department of Surgery, Patan Academy of Health Sciences, Lagankhel, Lalitpur, Nepal

**Keywords:** *neonate*, *sacrococcygeal teratoma*, *surgical excision*

## Abstract

Sacrococcygeal teratoma is a rare tumour of the coccyx that contains tissues from ectoderm, mesoderm and endoderm. It has a female preponderance with an incidence of 1 in 40,000 live births. Its prognosis depends on the tumour size, weight, APGAR scores, age at presentation, intraoperative injury and malignant potential. We report a case of Sacrococcygeal teratoma in a female neonate of birth weight 3.37 kgs, born to a 34 years primigravida. The tumour comprised about 1/3rd of the body weight which created hemodynamic challenges to the new-born. Successful surgical management of the tumour was done with a multidisciplinary team approach.

## INTRODUCTION

Sacrococcygeal teratoma (SCT) is the most common extra-gonadal germ-cell tumour in neonates and infants. The tumour develops either from the tip of the sacrum, protruding outwards from the buttocks or within the pelvic cavity. This tumour, having an incidence of 1 in 40,000, is derived from all three embryonic germinal layers and is more common in girls with predominance of 3-4:1.^1^ Though rare but these teratomas are more likely to be malignant in the adult population.^2^ It is diagnosed during antenatal care (ANC) visits, with ultrasonography in second trimester.^3^ However, the antenatally diagnosed teratoma has three times higher mortality rate than postnatally diagnosed teratoma.^4^ Surgical resection is the mainstay of treatment.^1^

## CASE REPORT

A female neonate was borne to a 34 year, primigravida with no known comorbidities via elective lower segment caesarean section at 36+^5^ weeks of gestation (WOG), the indication being a prenatally diagnosed congenital anomaly in the sacrococcygeal region. Her ultrasound scan done at 29+^1^ WOG showed amniotic band and swelling in the sacrococcygeal region, which was an exophytic and heterogenous mass, most likely a sacrococcygeal teratoma (Altman type II). After that, neurosurgery consultation was done and they advised for definite surgical management following delivery of the baby following a proper perinatal counselling. MRI of the fetus was done at 30^th^ WOG which showed complex cystic exophytic lesion of approximately 10.3 cm × 7.9 cm × 7.4 cm dimension along the sacrococcygeal region, with a small presacral extension, without significant pelvic extension.

Serial USG was done at subsequent ANC visits, which exhibited gradual growth of the swelling along with the fetus. There was no evidence of hydrops fetalis or fetal cardiac failure as fetal middle cerebral arterial peak systolic velocity (MCA-PSV) was within normal range at all times.

At birth, the weight of the baby was 3.37 kg with APGAR Score of 8/10 and 9/10 at one and five minutes of life, respectively. The congenital anomaly of a sacrococcygeal swelling with overlying skin defect, overlying hair and appendages was observed. Within the first few hours of life, neurosurgery and anaesthesiology consultations were made. Some imaging investigations like MRI whole neuroaxis (brain and spine), and echocardiography were done. Besides patent foramen ovale with a left to right shunt and mild tricuspid regurgitation in echocardiography, there weren't other associated anomalies.

**Figure 1:**
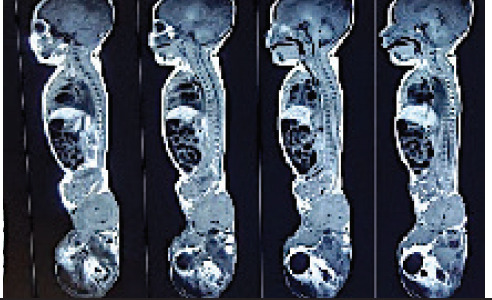
A 16.5×10.2×6.0 cm multiloculated cysto- solid mass arising from the lower vertebral end involving sacrococcygeal region with predominantly extra-fetal portion and small presacral component

MRI showed multiloculated cysto-solid mass of 16.5 cm × 10.2 cm × 6.0 cm dimension arising from the lower vertebral end involving the sacrococcygeal region with predominantly extra-fetal portion and a small presacral component containing multiple cysts and fat component without restricted diffusion (Figure 1).

Following these consultations and necessary preoperative investigations, surgery was planned.

**Figure 2:**
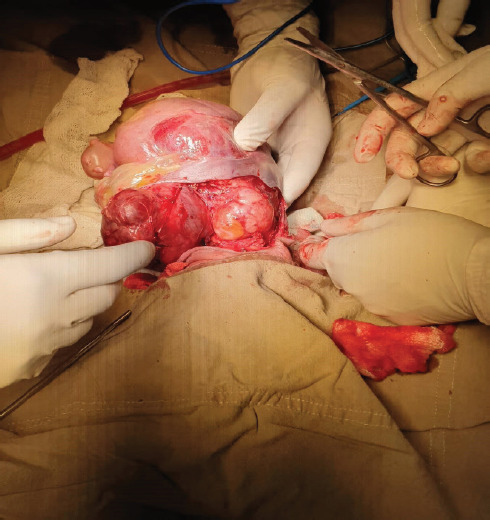
The tumour (approximate dimension: 18×12×9 cm) being separated from the adjacent tissues using the tumour capsule as a guide

With a multidisciplinary team, surgery was done with total excision of the sacrococcygeal swelling with removal of coccyx with soft tissue reconstruction on the third day of life. The swelling measured 1.2 kg with dimensions of 18 cm × 12 cm × 9 cm (Figure 2).

**Figure 3:**
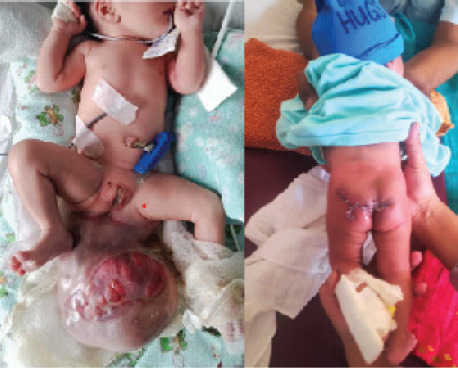
Newborn on 1st day of life (Right) presenting with a large mass in the sacrococcygeal region and newborn on 20th day of life after surgery (Left)

Following surgery, the neonate stayed in NICU for two days. Feeding was started from the first post-operative day. There were spontaneous movements of lower limbs. There was normal passing of meconium, faeces and urine following surgery. Wound dehiscence occurred on 4th day of the surgery and re-closure was done on 20th day of life of the neonate, following care in prone positioning with head up inclined position, frequent clearing of soilage, regular dressings and proper antibiotic coverage (Figure 3).

## DISCUSSION

Sacrococcygeal teratomas originate from the area of primitive streak called primitive knot or Hensen's node. The primitive streak appears as linear thickening in ectoderm at the caudal edge of bilaminar embryonal disc. As the mesoderm rapidly proliferates, primitive streak comes to lie more caudally where remnant of Hensen's node descends to the tip of coccyx or its anterior surface. Normally, the primitive streak undergoes degenerative changes and disappears, but primitive streak remnants may persist and give rise to a tumour known as sacrococcygeal teratoma. These tissues are derived from ectoderm, mesoderm and endoderm.^1^

In our case, the teratoma was of Altman type 2.^5^ During perioperative procedure, there can arise pre-operative, intra-operative and post-operative complications. These include massive bleeding, rupture, high output cardiac failure, disseminated intravascular coagulation, anaemia, wound infection, etc. Similarly, long term complications include tumour recurrence, malignant transformation, bowel and bladder dysfunction, lower limb palsy and cosmetically unacceptable scarring.^6^ In our case, we encountered post-operative wound dehiscence and infection as a challenge, which we could successfully overcome.

For tumour lesions outside the pelvis, it is reasonable to consider caesarean section depending on the size in order to avoid tumour rupture, haemorrhage and delivery difficulty and improve the prognosis, especially in the case of large tumours. The vital prognosis, however, is affected by many other factors, making it hard to conclude that it can be improved by caesarean section alone. Although interventional radiology (IVR) for sacrococcygeal tumour facilitates its removal and may reduce the amount of bleeding at the time of removal, only a few cases of IVR have been documented, and a skilled technique is required for the procedure. Therefore, we recommend that the feasibility of the procedure at each facility be thoroughly examined before its application. Although the risk of recurrence of malignant teratomas is high, even mature or immature teratomas may develop malignant recurrence. To address possible complications, routine follow up is to be made in every three years. Alpha fetoprotein, Beta Human Chorionic Gonadotropin hormone and imaging modalities like ultrasound sonography and MRI can be done to know the recurrence or malignant potential of the teratoma.^6^

In a study by Isserman RS et al,^7^ focus was made on factors affecting perioperative mortality, where gestational age less than or equals to 30 weeks, non- cystic component and surgeries done on emergent basis were identified as significant factors. Our case had a mixture of cystic and non-cystic components, and despite this, the patient survived and made a good post-operative progress.

Derikx JP et al.^8^ conducted a study on different factors in relation with recurrence of sacrococcygeal teratoma and metastasis, and found significant association with immature and malignant histotype and incomplete resection. In our case, histopathology reports of the completely resected teratoma yielded a mature histotype. Their study didn't show a significant correlation between recurrence and removal of coccyx, that defied the conventional notion. However, in our case, we removed the coccyx also.

Maximum tumour diameter of 17 cm, tumour volume of 710 grams and more, 1-minute Apgar score <7, requirement for abdomino-sacral resection and intraoperative injuries were identified as significant factors affecting the long-term functional outcome of sacrococcygeal teratoma after resection in neonates and infants in a study done by Masahata K et al.^9^ While, the 1-minute Apgar score of our patient was above 7, there was no requirement for abdomino- sacral resection and no intraoperative injury, the maximum tumour diameter was 18 cm and the tumour volume was 1200 grams. So, we planned to follow up our patient for at least 3 years, with the first follow up being at 3 months of life. Then, we planned to look for occurrence of any functional disorder, and also for the serum alpha-fetoprotein levels, as the pre-operative values were above 500IU/mL.
